# Measles outbreak in Western Uganda: a case-control study

**DOI:** 10.1186/s12879-021-06213-5

**Published:** 2021-06-22

**Authors:** Abel Wilson Walekhwa, Moses Ntaro, Peter Chris Kawungezi, Chiara Achangwa, Rabbison Muhindo, Emmanuel Baguma, Michael Matte, Richard Migisha, Raquel Reyes, Peyton Thompson, Ross M. Boyce, Edgar M. Mulogo

**Affiliations:** 1 Department of Community Health, Faculty of Medicine, Mbarara University of Science & Technology, P.O. BOX 1410, Mbarara, Uganda; 2 Department of Public Health and Hygiene, University of Buea, P.O. Box 63, Buea, Cameroon; 3 Department of Physiology, Faculty of Medicine, Mbarara University of Science & Technology, Mbarara, Uganda; 4 Department of Medicine, Division of Hospital Medicine, University of North Carolina at Chapel Hill, Chapel Hill, NC 27599 USA; 5 Department of Pediatrics, Division of Infectious Diseases, University of North Carolina at Chapel Hill, Chapel Hill, NC 27599 USA; 6 Department of Medicine, Division of Infectious Diseases, University of North Carolina at Chapel Hill, Chapel Hill, NC 27599 USA

**Keywords:** Measles, Outbreak, Immunization, Epidemiology, Uganda

## Abstract

**Background:**

Measles outbreaks are prevalent throughout sub-Saharan Africa despite the preventive measures like vaccination that target under five-year-old children and health systems strengthening efforts like prioritizing the supply chain for supplies. Measles immunization coverage for Kasese district and Bugoye HC III in 2018 was 72 and 69%, respectively. This coverage has been very low and always marked red in the Red categorization (below the national target/poor performing) on the national league table indicators. The aim of this study was to assess the scope of the 2018–2019 measles outbreak and the associated risk factors among children aged 0–60 months in Bugoye sub-county, Kasese district, western Uganda.

**Methods:**

We conducted a retrospective unmatched case-control study among children aged 0–60 months with measles (cases) who had either a clinical presentation or a laboratory confirmation (IgM positivity) presenting at Bugoye Health Centre III (BHC) or in the surrounding communities between December 2018 and October 2019.. Caregivers of the controls (whose children did not have measles) were selected at the time of data collection in July 2020. A modified CDC case investigation form was used in data collection. Quantitative data was collected and analyzed using Microsoft excel and STATA version 13. The children’s immunization cards and health registers at BHC were reviewed to ascertain the immunization status of the children before the outbreak.

**Results:**

An extended measles outbreak occurred in Bugoye, Uganda occured between December 2018 and October 2019. All 34 facility-based measles cases were documented to have had maculopapular rash, conjunctivitis, and cough. Also, the majority had fever (97%), coryza (94.1%), lymphadenopathy (76.5%), arthralgias (73.5%) and Koplik Spots (91.2%) as documented in the clinical registers. Similar symptoms were reported among 36 community-based cases. Getting infected even after immunized, low measles vaccination coverage were identified as the principal risk factors for this outbreak.

**Conclusion:**

Measles is still a significant problem. This study showed that this outbreak was associated with under-vaccination. Implementing a second routine dose of measles-rubella vaccine would not only increase the number of children with at least one dose but also boost the immunity of those who had the first dose.

**Supplementary Information:**

The online version contains supplementary material available at 10.1186/s12879-021-06213-5.

## Background

Measles is a highly infectious disease caused by the measles virus, which has an attack rate greater than 90% among susceptible persons [1]. Classic symptoms include fever, cough, coryza, conjunctivitis, and maculopapular rash. The rash appears as red spots on the skin, typically 3 to 5 days after the development of other symptoms [2, 3]. Complications of the disease, which include ear infections, pneumonia, and encephalitis, are common and can be fatal, especially in malnourished or immune compromised children [4].

With an almost 300% increase in cases reported between 2016 and 2018, measles continues to represent a significant public health challenge in sub-Saharan Africa [5]. Estimates suggest that upwards of 14 million children under five years of age live in “cold spots,” defined as highly endemic/high danger areas, across the region, and 8–12 million children remain unvaccinated [6]. In 2018 alone, models suggested that measles affected nearly ten million children and resulted in 140,000 deaths globally [7]; the African region was among the most impacted regions with 1,759,000 total cases and 52,600 deaths [8]. Unfortunately, recent trends continue to show increases in all regions [5]. This increase was particularly pronounced in the WHO Africa Region, which recorded a 700% increase over the first four months of 2019 [9], highlighting that measles outbreaks remain a major public health challenge.

In Uganda specifically, the measles situation has remained relatively stagnant. For example, in 2018, 46 districts reported measles outbreaks [10, 11]. The current Uganda National Expanded Program on Immunization (UNEPI) schedule recommends a single measles vaccination (MCV) at nine months of age [12]. This is in contrast to WHO recommendations, which suggest that reaching all children with two doses of measles vaccine should be the standard for all national immunization programs [13]. Furthermore, the WHO states that in countries with ongoing transmission in which the risk of measles mortality remains high, the first dose (MCV1) should be given at nine months of age and a second dose should be given between 15 and 18 months of age [14, 15].

From December 2018 through October of 2019, Bugoye Health Centre III (BHC), located in Kasese District of southwestern Uganda, reported a large number of suspect measles cases. Eight clinical samples were submitted to the Uganda Virus Research Institute (UVRI) for serological testing. Five of these samples were confirmed (i.e. IgM positive) and the clinical staff were subsequently instructed to use clinical diagnosis for any additional cases. All cases were documented pending further investigation according to BHC records [16].

Therefore, the objective of this study was to investigate the scope of and the demographic, clinical, and geographic risk factors associated with the 2018–2019 measles outbreak in Bugoye subcounty.. Specifically, we focused on children aged 0–59 months, given the higher risk of complications and mortality. Our goal was to inform policymakers, including District Councilors and sub-county leadership, of the risk factors associated with measles outbreaks in order to influence resource allocation and guide potential interventions at the District Health Office level.

## Methods

### Study setting

**Fig. 1:**
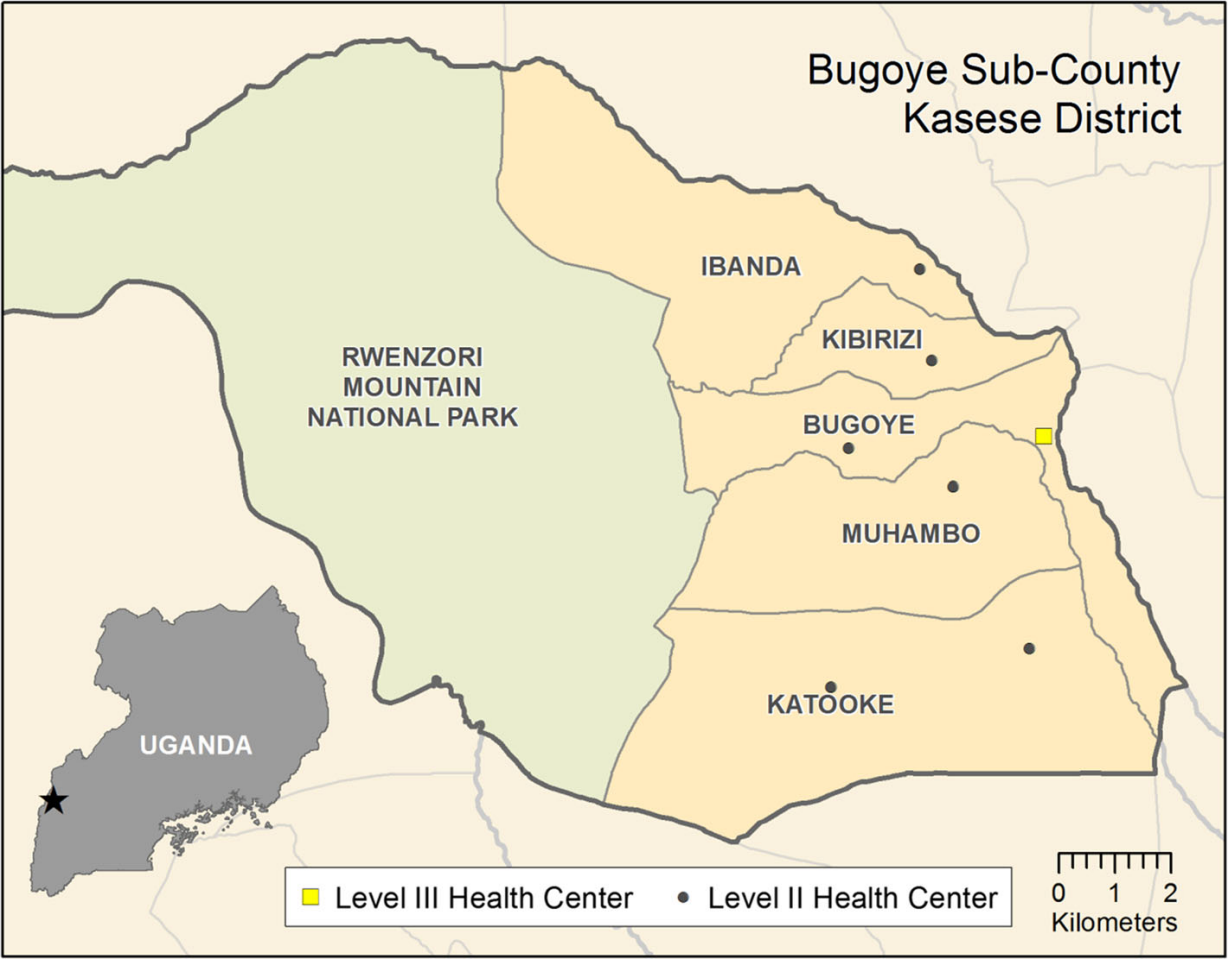
Map of Bugoye Subcounty, Kasese District

The study was conducted in Bugoye sub-county, Kasese district in Southwestern Uganda (Fig. 1). The sub-county was selected following reports of measles cases presenting to local health centres. Bugoye spans a rural, highland area of approximately 55 km^2^, which is characterized by deep river valleys and steep hillsides with elevations up to 2000 m. The sub-county is comprised of five parishes and thirty-five villages with a population of approximately 50,000, one-quarter of whom are children under 5 years of age [17]. The main economic activity is agriculture with most households engaged in subsistence farming.

There are eight health facilities in the sub-county including six level II and two level III health centres, one of which is a private, not- for- profit facility. Bugoye Level III Health Centre (BHC) is the only public level III health facility in the sub-county. Level III Health Centres are staffed by a senior clinical officer, a clinical officer, laboratory technicians, health assistants, nurses, and midwives. Available services include routine antenatal care, an immunization clinic, a general outpatient clinic, an antiretroviral clinic for patients with HIV, and small inpatient and maternity wards. Level II health centres provide immunization services, antenatal care, and outpatient clinics, but do not offer inpatient services or HIV care. The health facilities offer immunizations services through two models; static (facility based) during week days (Monday to Friday) then outreaches (community based) that are organized one per month. Current Ugandan immunization guidelines recommend measles immunization with a single dose at the age of nine months [18].

### Study design

#### Case-Control investigation

For the purposes of the investigation, WHO measles case definitions of both suspected and confirmed were used [19]. Facility-based cases were defined as children less than or equal to five years of age who met the following criteria:

1. We defined a suspected case as fever (temperature ≥ 37.5 °C) and generalized skin rash lasting for three or more days with at least one of the following: cough, coryza and conjuctivitis in any child aged 0–60 months presenting at Bugoye health centre, or residing in Bugoye Sub-county between April 2018 to October 2019.
2. We defined a confirmed case as any suspected case with measles IgM antibodies.
3. A control was any child (aged 0–60 months) who did not have any rash April 2018 to October 2019 and was not living in the same household with a case- control, randomly selected from the neighbourhood at a ratio of 1:2.

All methods were carried out in accordance with relevant guidelines and regulations. The names and contact information of eligible cases had been previously recorded at BHC for further investigation. In collaboration with local community health workers, also known as Village Health Teams (VHTs) in Uganda, study staff contacted the caregivers of cases by visiting the households.. Study staff identified themselves to the caregiver and provided information about the objectives, methods, and risks/benefits of the study.

After consent was provided from caregivers, staff administered a modified case investigation form (Supplementary form X), which had been translated into the local language (Lukonzo). The latitude and longitude of participating households were recorded using a GPS application installed on mobile phones. Child immunization cards under possession of the caregivers and health registers at facilities were reviewed to verify immunization of participating children before the outbreak occurred. For the caregivers who did not have these cards or did not know the status of vaccination of their children, the research team verified the immunization status of the children from Immunization and delivery registers at BHC, where all the caregivers take their children for immunization. A total of 27 out of 39 cases whose vaccination status was uncertain were verified through this effort. The study team is cognizant of the mass measles-rubella (MR) vaccination campaign in Uganda and we cross-checked our data to ensure that the vaccination status was not confused with the latter. This was also aided by the different MR immunization cards (pink in color) that had been issued during the mass campaigns. The study team verified with the usual child immunization cards (white in color).

In the course of fieldwork, additional suspected cases were identified in the community. These children had experienced a similar clinical syndrome, but presented to health facilities other than BHC for evaluation. A total of 37 community-based cases were identified with the help of the VHT and local council leaders. A review of the children’s measles status was done by cross- checking records in the health facilities where the children had been taken for treatment. Records were screened to identify those who met the eligibility criteria; one case was excluded while 36 cases met inclusion criteria. All 36 cases had blood samples taken for serological analysis and reported to Kasese District. The decision to include these community-based cases in the analysis was made after consulting with the academic mentor and study team at large. In households where there were multiple children, an eligibility criterion was used to identify those fit. In circumstances where multiple children met the criterion, a simple random sampling technique was applied by assigning random unique numbers to the children and later selecting one of them.

The health centers from whence these cases are reported are also public health facilities and report their immunization activities to the office of the Kasese district health officer. Controls for this additional group were defined as those who met the eligibility criteria, were from the same village and within two years of age of the identified case,, but were not suspected to have measles.

Study staff completed similar procedures, including enrollment, caregiver interview, and review of immunization cards, for controls as they did for cases. Based on the number of eligible facility-based cases, we estimated the anticipated number of the controls to be 100. This was calculated using the known number of cases (*n* = 50) reported to the national surveillance system by BHC, with a 2:1 ratio of controls to cases and a significance level set to 5%, the least extreme odds ratio to be detected was 0.44 at 100% study power. The computation was done by OpenEpi software for Unmatched case control study [20].

### Statistical analysis

Participant data was recorded in the Open Data Kit (ODK) enabled database [21] and analyzed using STATA Version 13 (Stata Corp, College Station, TX). We summarized the demographic and clinical characteristics of participants, including caregivers, and compared them between cases and controls. The reported cases and caregivers with missing information where not included during analysis of the data to ensure complete case analysis. For example, for the cases whose caregivers were not found at home and several attempts made to reach the caregivers in vain.

Two-sided chi-square tests for association were computed to detect relationships between categorical variables such as vaccination status, living conditions, age of the children, occupation of the caregivers and the primary outcome of a measles diagnosis. The significance was set at a *p*-value level of 0.05.

Explanatory variables that were hypothesized to have an association with the primary outcome diagnosis were analyzed using univariate logistical regression. Variables that were statistically significant in univariate models with a pre-specified *p*-value of < 0.2 were included in the subsequent multivariate analysis and a resulting *p* < 0.05 taken to be statistically significant in the final model [22].

An epidemiological curve represented with a bar graph was also drawn using Microsoft Excel 13 (Redlands, WA) to show the trend of the confirmed cases per week.

## Results

**Fig. 2:**
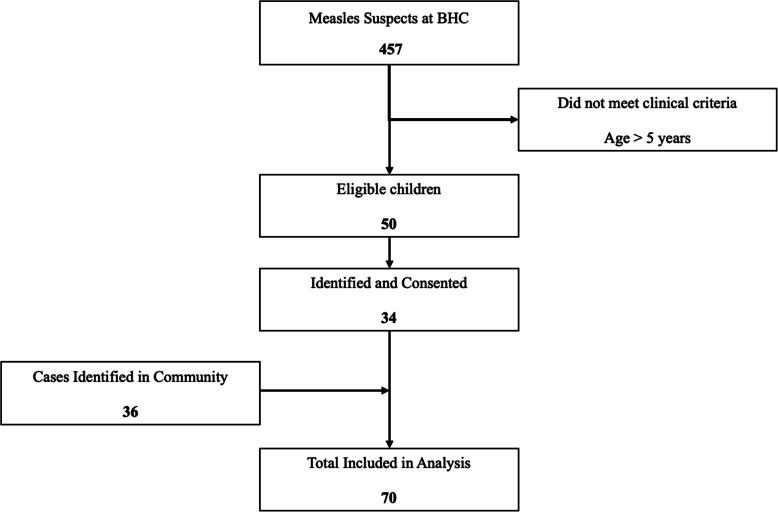
Flowchart detailing screening and enrollment of participating cases

Study activities were conducted from June 15th to August 7th 2020. Review of clinical registers showed that 457 children were considered measles cases during the period of investigation. A total of 50 (10.9%) of these diagnosed with measles were below five years of age thus were determined to be eligible for the study (Fig. 2). Study staff were able to locate and consent 34 of 50 (68.0%) eligible facility-based cases. A total of 79 controls were matched with those known by the health care system in a 2:1 ratio. Field staff were approached by caregivers of another 36 suspect cases who had been seen in other health facilities or managed in the community. After review of the clinical histories, these additional 36 children were included in the study. A number of these community-based cases occurred in the same household. In these cases, a single control was selected for the household rather than for each child, resulting in the selection of another 21 controls.

**Fig. 3:**
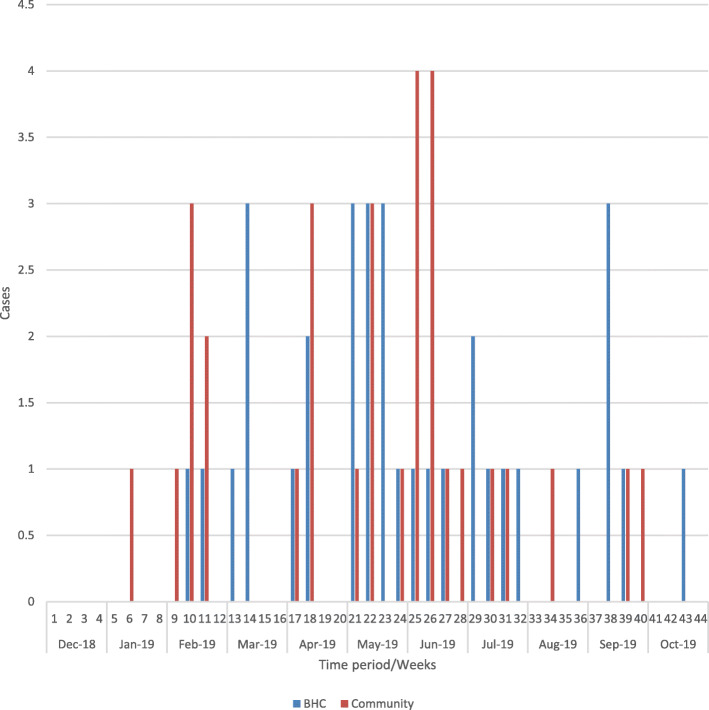
An epidemic curve for measles outbreak in Bugoye Subcounty

Review of the health facility data showed that the first confirmed measles case was reported in December 2018 with incident cases continuing through October 2019 as represented by an pidemic curve for the measles outbreak in Bugoye subcounty (Fig. 3). All facility-based cases were documented to have maculopapular rash, conjunctivitis, and cough. Fever (97%), coryza (94.1%), and Koplik Spots (91.2%), lymphadenopathy (76.5%) and arthralgias (73.5%) were documented in the clinical registers. Similar symptoms were reported among the 36 community-based cases (Table 1).

**Table 1:** Reported clinical symptoms of facility- versus community-based cases

Clinical Symptom	Facility Based	Community Based
Fever	33 (97.1%)	36 (100%)
Rash	34 (100%)	36 (100%)
Maculopapular Rash	34 (100%)	35 (97.2%)
Conjunctivitis	34 (100%)	36 (100%)
Cough	32 (94.1%)	36 (100%)
Coryza	32 (94.1%)	36 (100%)
Koplik Spots	31 (91.2%)	33 (91.7%)
Lymphadenopathy	26 (76.5%)	31 (86.1%)
Arthralgia	25 (73.5%)	28 (77.8%)

Demographic characteristics of cases and controls are presented in Table 2. There were more cases among females then males (57.1% vs 42.9%, *p* = 0.02). The majority of the case households were located in rural areas 57 (81.4%) with only 13 (18.6%) residing within the confines of a more densely-populated trading centre. Most case households were located within one kilometer of BHC (39 of 50, 78%). Controls generally had similar demographic characteristics with the exception that a higher proportion of controls were male (61.0% vs. 39.0%, p = 0.02).

**Table 2:** Demographic characteristics of the cases and controls

Characteristic/factor	Cases	Controls	*p*-value
Total (n,%)	70 (100.0)	100 (100.0)	–
Age of child (Mean 2.8, SD 1.34)	Mean = 3.2, SD 2.65	Mean = 2.6, SD 1.75	
1 year	12 (17.1)	26 (26.0)	0.14
2 years	13 (18.6)	26 (26.0)	
3 years	11 (15.7)	19 (19.0	
4 years	21 (30.0)	18 (18.0)	
5 years	13 (18.6)	11 (11.0)	
Sex of child			
Male	30 (42.9)	61 (61.0)	0.02
Female	40 (57.1)	39 (39.0)	
Vaccine status			
Vaccinated	48 (68.6)	80 (80.0)	0.09
Unvaccinated	22 (31.4)	20 (20.0)	
Caregiver Education			
Primary School	40 (57.1)	62 (62.0)	0.80
Secondary School	21 (30.0)	28 (28.0)	
University	4 (5.7)	6 (6.0)	
None / Illiterate	5 (7.1)	4 (4.0)	
Type of locality			
Trading Center	13 (18.6)	15 (15.0)	0.54
Rural	57 (81.4)	85 (85.0)	
Distance moved to facility			
< 1 km	39 (55.7)	49 (49.0)	
> 1 km but < 3 km	26 (37.1)	41 (41.0)	0.68
> 3 km but < 5 km	3 (4.3)	8 (8.0)	
> 5 km	2 (2.9)	2 (2.0)	

The vast majority of cases were HIV-unexposed at birth as reported by the caregivers and within the normal range of height/weight (Table 2). Approximately two-thirds of all cases (48 of 70, 68.6%), including 23 (67.7%) facility-based cases and 25 (69.4%) of community-based cases, had documentation of measles vaccination prior to clinical illness, while one-third (22 of 70) were not previously vaccinated. Among cases who were vaccinated, the median duration between vaccination and disease onset was 885 days (IQR 575–1340). A higher proportion of controls (80 of 100, 80%) reported receiving measles vaccination (*p* = 0.09). Controls had similar baseline HIV and nutritional characteristics.

In the univariate logistic regression model, sex (female), age (≥ 4 years), and vaccination status met the pre-specified level of significance and were included in the multivariate model (Table 3). In the multivariate model, female sex (aOR 1.98, 95% CI 1.04–3.78, *p* = 0.04) and an age of ≥4 years (aOR 3.14, 95% CI 1.18–8.38, *p* = 0.02) were most strongly associated with disease. Children without previous measles vaccination were at slightly more than double the odds of disease, but this association did not meet statistical significance (*p* = 0.06).

**Table 3:** Logistic regression analysis of disease correlates

Variable	Unadjusted Model			Adjusted Model*		
	OR	95% CI	***p***-value	aOR	95% CI	***p***-value
Gender (Female)	2.09	1.12–3.88	0.02	**1.98**	**1.04–3.78**	**0.04**
Age (Years)						
1 year or less		REF			REF	
2 years	1.08	0.42–2.81	0.87	1.21	0.45–3.26	0.71
3 years	1.25	0.46–3.44	0.66	1.32	0.47–3.76	0.60
4 years	2.53	1.00–6.40	0.05	**3.14**	**1.18–8.38**	**0.02**
5 years	2.56	0.89–7.35	0.08	2.85	0.95–8.55	0.06
Caregiver Education						
Primary		REF			–	
Secondary	1.16	0.58–2.32	0.67		–	
Tertiary	1.03	0.27–3.89	0.96		–	
None/Illiterate	1.94	0.49–7.65	0.35		–	
Distance to Clinic	0.70	0.29–0.61	0.48		–	
Less than 1 km		REF			–	
1–3 km	0.80	0.41–1.52	0.49		–	
3–5 km	0.47	0.12–1.90	0.29		–	
More than 5 km	1.26	0.17–9.33	0.82		–	
Unvaccinated	1.83	0.91–3.70	0.09	2.06	0.97–4.39	0.06

* Variables that were significant in univariate models with a pre-specified *P*-value of < 0.25 were included in the subsequent multivariate analysis

## Discussion

Our investigation documents an extended outbreak of measles that occurred among young children in a rural western Uganda. The outbreak was likely sustained by low immunization rates in the community, estimated to be only 68.9% among cases, which falls far below the critical threshold of > 95% required to interrupt transmission. However, these results also call into question the efficacy of a single-dose immunization strategy given the high proportion of cases that occurred in vaccinated children.

The measles outbreak in Bugoye sub-county occurred between December 2018 and October 2019. Notably, this was a period of renewed conflict in the neighboring Democratic Republic of Congo (DRC) that resulted in significant displacement across borders in this region [23]. Measles outbreaks were ongoing in the DRC during this time and migration may have facilitated geographic spread to the neighboring Kasese District. The largest number of cases occurred during the traditional dry season (February – May) in Uganda, when weather conditions were dusty and this may have further accelerated the spread of the disease [24].

The impact of low vaccination rates in the communities is far-reaching in contributing to the outbreak of such a vaccine-preventable disease since those missed opportunities tend to accumulate over time. This study established that the low vaccination rate is among the factors that could have contributed to the measles outbreak in BHC. The immunization coverage can be improved through maintained vaccine stocks aided by prior planning, enhancing the outreach activities through increased funding, identifying the children who missed the vaccination through review of health facility records, reminding caregivers through social mobilization using the existing structures of the VHTs, involving more technical health workers in immunization activities, increasing the days for static immunization at BHC to include weekend days, and developing an immunization emergency preparatory plan for rainy seasons. Our study findings are in agreement with other measles outbreaks in Uganda [25, 26].

Our findings also demonstrated that most children contracted measles years after vaccination. This would suggest the single dose is not sufficient to confer lasting immunity. Further evidence of this was shown in the regression analysis where children ≥4 years of age were at the highest risk. This is in agreement with other studies conducted in other parts of the world, supporting the WHO position recommending a second dose at 15–18 months of age [27–29]. Furthermore, previous studies have also shown that insufficient immunity after one dose of measles vaccine given at 9 months can be due to vaccine failure and/or having maternal antibodies present that dampen the response, more so than waning immunity [30]. A second routine dose of vaccine would address both of these risk factors, by increasing the likelihood of a child receiving at least one dose and also by boosting the immunity of children who received the first dose [31]. Our recommendations will help the Ministry of Health to increase the coverage of measles- rubella vaccination, which will in turn help to mitigate outbreaks of rubella, which has now also been shown to be endemic across the country and the region [32].

Our study has a number of strengths including the rigorous case definition, verification of immunization status through multiple sources, and double methods of data collection which enabled us to understand some salient issues during key informant interviews.

Our investigation also has limitations that largely result from the retrospective nature of the study and possible selection bias at the household level in that some children could have been taken to the privately owned clinics, especially those who were severely sick. These cases could have been missed in the healthcare system, thus not being selected to participate in the study. Some of the caregivers of both the cases and controls had misplaced their immunization cards and this affected the efforts of comparing the data at the health facility and the child immunization cards.

Our study was conducted just after the mass Measles Rubella (MR) vaccination campaign, which hindered retrospective serological investigation of the cases and may have affected our results. Also, there was a small sample of children who were tested for IgM positivity at UVRI for confirmation of measles, with many children instead diagnosed clinically. Furthermore, due to a limited research budget, the study team only included all controls in households that were geographically close to those of cases. Finally, these results may have suffered from recall bias since the study team based some results on verbal responses from the caregivers.

## Conclusion

Measles remains a significant problem in Uganda. This study suggests that the 2018–2019 outbreak was driven by low vaccination rates and waning and/or impartial immunity following a single immunization. These results strongly support further outreach to underserved communities, and also provide additional evidence for policy change.

## Supplementary Information

**Additional file 1.**

## Data Availability

All data supporting our findings are contained in the paper. There are no restrictions to data sources, however, details of the full data may be accessed through Mr. Abel Wilson Walekhwa (corresponding author), Department of Community Health, Mbarara University of Science and Technology, PO Box 1410, Mbarara, Uganda, email:wabelwilson@gmail.com, Tel: + 256752206865.

## Abbreviations

BHC: Bugoye Health Centre III
MOH: Ministry Of Health
UNEPI: Uganda National Expanded programme on Immunisation
UVRI: Uganda Virus Research Institute
VHT: Village Health Teams
WHO: World Health Organization
