# Supplementary material for: Measles outbreak in Western Uganda: a case-control study
Source: BMC Infect Dis. 2021 Jun 22;21:596. doi: 10.1186/s12879-021-06213-5 (PMC8220759; doi:10.1186/s12879-021-06213-5)
Supplement: Supplementary file 1 — Additional file 1. [file 12879_2021_6213_MOESM1_ESM.docx]

**Measles Outbreak in Western Uganda: A Case-Control Study**

Walekhwa Abel Wilson*^1^, Moses Ntaro^1^, Peter Chris Kawungezi^1^, Chiara Achangwa ^3^, Rabbison Muhindo^1^, Emmanuel Baguma^1,^ Michael Matte^1^_,_ Richard Migisha^4^, Raquel Reyes^5^, Peyton Thompson^6^, Ross M. Boyce^1,2^ and Edgar M. Mulogo^1^_._

Supplementary form X: **Investigation Form, modified January 23,2008)**

**Part A: Patient's Information (From Child’s Immunization Card)**

1. Age of child:………………………….
2. Gender: 1-Male 2- Female

**Vaccination History**

i) Type of vaccine 1-measles 2- Rubella 3- Measles Rubella 4- Measles Mumps Rubella

ii) Number of doses 1- Zero dose 2- One dose 3-Two doses 4-Three doses 99-unknown

iii) Date of last dose:........./............/..........

iv) Source of vaccination information 1-vaccination card 2-Health service record 3-verbal

**Clinical Data, Follow up and Treatment**

i) Fever 1-yes 2- No 99-Unknown, if yes, Temperature........^0^C, Date of onset ....../,...../......

ii) Rash? 1-Yes 2-No 99- Unknown. If Yes, Duration of the rash in days......, Type of rash 1- maculopapular 2- Vacuolar 88-other 99-Unknown

iii) Conjunctivitis? 1-yes 2- No 99-Unknown

iv) Cough? 1-yes 2- No 99-Unknown

v) coryza? 1-yes 2- No 99-Unknown

vi) Koplik spots? 1-yes 2- No 99-Unknown

vii) Lymphadenopathy? 1-yes 2- No 99-Unknown

viii) Arthralgia? 1-yes 2- No 99-Unknown

ix) Is the patient pregnant? 1-yes 2- No 99-Unknown

x) if yes, weeks of the pregnancy, 0-42? .................,

xi) The place where the birth is likely to take place:......................

xii) Was the patient hospitalized? 1-yes 2- No 99-Unknown

xiii) If yes, which hospital?..................., Date of admission...../...../...... admission Number:.......

xiv) Death? 1-yes 2- No 99-Unknown

xv) if yes, date of death:...../...../..... Primary cause of death..........................

xvi) What was the HIV status of your child in 2018?

1. HIV+ 2- HIV- 9-Not sure

**Nutrition Status**

xvii) Did your child ever suffered from malnutrition in 2018?

1-Yes 2-NO 99- I don’t recall 9- Not sure

xviii) If yes, which condition?

1-Kwashiorkor 2-marasmus 3- rickets 5- scurvy 8-others (specify) ...........................

8-others (specify)...........................

**Part B: Household/caregiver parameters**

1. Age of the caregivers (in years)…………
2. Gender: 1-Male 2- Female
3. Marital status

1-Single 2-Married 3- Divorced 4- widow 5-widower 8-others(specify)………..

1. Religion

1-moslem 2- catholic 3- protestant 4-Pentecost 8-others(specify)…………..

1. Level of Education

1-Completed Primary 2-Secondary (didn’t finish) 3- Finished O’ level 4- Completed A’ level 5-Tertiary

1. Occupation

1-Housewife 2-VHT 3- LC 4- Teacher 5- Peasant 8-others(specify)……....

1. Monthly income

1-<5,000/= 2- 5,000/= -30,000/= 3- 30,000/= - 50,0000/= 4- > 50,000/=

1. Address:

Village...........................Parish:..............................Subcounty:.........................District:..............Landmarks to locate the house..........................................................................

Telephone:.............................

Type of locality 1-Urban 2- Peri urban 3- Rural

1. Observe type of living house,

1-Permanent, well finished 2-Permanent,not finished 3-Semi permanent not dusty 4-Semi permanent, dusty 5-Temporary,grass thatched, dusty 8-Others(specify)……….

x) How many family members stay in this house?

1-Less than 4 2- between 4 and 8 between 3-8 and 12 4- more than 12

xi) How many are under five years?......................................

xii) Patient's date of birth......./........../............ or age:..... Years............Months..........

xiii) Distance to the neighbouring health facility

1-<1km 2-more than 1km but less than 3 3-more than 3 km but less than 5km 4->5km

xiv) What transport do you use to the health facility?

1. Walk 2-motorcycle (Bodaboda) 3-Bicycle 4- Motor vehicle 8-Others(specify)..................

xv) What do you think about immunization?

1- Good 2-Not good 9-Not sure

xvii) If good, Give some importance of immunization according to you

........................................................................................................

..........................................................................................................

............................................................................................................

xviii) Where did you deliver your child from

1-Home 2- Traditional birth attendant 3-Health facility 8-Others (specify).........................
